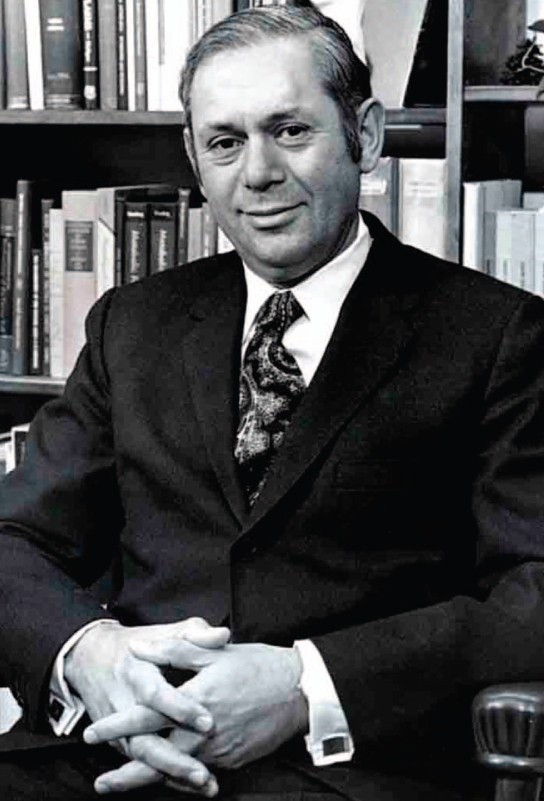# Paul Kotin, MD: 1916–2008

**Published:** 2008-09

On 12 May 2008, Paul Kotin, MD, pioneer environmental health physician-scientist and NIEHS director from 1966 to 1971, passed away in Laguna Beach, California.

A pathologist by training, Kotin received his MD in 1939 from the University of Illinois, and later served in the U.S. Army Medical Corps during World War II, followed by private practice and appointments at Los Angeles County Hospital and the University of Southern California. He joined the National Cancer Institute (NCI) in 1962, where he became director of the Division of Cancer Etiology at the NCI. In November 1966, the NIEHS—originally named the National Institutes of Health (NIH) Division of Environmental Health Sciences—was officially established, with Kotin as head. Under his direction, the NIEHS achieved institute status in 1969 as the ninth institute in the NIH, with Kotin as the first director and a budget of $17.8 million.

Sandy Lange, a staff assistant during Kotin’s tenure at NIEHS, as well as a former director of both the Office of Communications and Public Liaison and the National Toxicology Program Office of Liaison and Scientific Review, recalls how Kotin’s vision of the institute’s mission laid the groundwork for its growth into a definitive source of public health information: “Times for the NIEHS when Paul Kotin started as the first director . . . were challenging and exciting. He was totally committed and dedicated to educating and translating the mission and role of the new division to the scientific community at large, including the other NIH institutes and other government agencies, Congress, industry, labor, and the public interest groups. He laid the foundation for university-based centers of excellence and training programs when there were no environmental health programs within the universities. He and Hans Falk [his Scientific Director] laid the scientific foundation for the division. He built partnerships and did battle as was necessary to protect the role and location of this new program as a centerpiece within the federal government. During the years following his tenure at NIEHS, we spoke relatively often, and he followed the institute’s growth with interest. He had great respect for the institute’s accomplishments, its leadership, and its contributions—he often said to me that it takes one type of leadership to start a program and another to build it. He praised the work of the leaders and the scientists who followed. He was that kind of man; and it was truly an honor to work with him. NIEHS owes much to his early work.”

In his later career, Kotin served as dean of the School of Medicine, vice president for Health Sciences, and provost at Temple University in Philadelphia (1971) and senior vice president for Health, Safety and Environment for the Johns-Manville Corporation in Denver (1974). After retiring in 1981, he remained active, serving on a National Academy of Sciences oversight committee for the Department of Energy’s management of the U.S. nuclear stockpile from 1988 to 1990.

Kotin was widely regarded as an international expert on environmentally caused lung diseases, especially those caused by toxic substances such as asbestos and beryllium, and received a number of awards for his contributions to public health, including the U.S. Department of Health, Education and Welfare Superior Service Award and Distinguished Service Award and the Knudsen Award from the American Occupational Medicine Association.

He is survived by his wife Pauline Kotin, two sons, four grandchildren, and two great-grandchildren.

## Figures and Tables

**Figure:**